# Inflammatory burden, lifestyle and atherosclerotic cardiovascular disease: insights from a population based cohort study

**DOI:** 10.1038/s41598-023-48602-7

**Published:** 2023-12-08

**Authors:** Benjamin Bay, Christopher Blaum, Caroline Kellner, Ramona Bei der Kellen, Francisco Ojeda, Julia Waibel, Natalie Arnold, Christian-A. Behrendt, David L. Rimmele, Goetz Thomalla, Raphael Twerenbold, Stefan Blankenberg, Birgit Zyriax, Fabian J. Brunner, Christoph Waldeyer

**Affiliations:** 1 Department of Cardiology, University Heart and Vascular Center Hamburg, University Medical Center Hamburg-Eppendorf, Martinistrasse 52, 20246 Hamburg, Germany; 2 German Center for Cardiovascular Research (DZHK), Partner Site Hamburg/Kiel/Lübeck, Hamburg, Germany; 3 Center for Population Health Innovation (POINT), University Heart and Vascular Center Hamburg, University Medical Center Hamburg-Eppendorf, Hamburg, Germany; 4 Department of Neurology, University Medical Center Hamburg-Eppendorf, Hamburg, Germany; 5 Research Group Preventive Medicine and Nutrition, Midwifery Science-Health Care Research and Prevention (IVDP), University Medical Center Hamburg-Eppendorf, Hamburg, Germany

**Keywords:** Cardiology, Risk factors, Cardiovascular diseases, Vascular diseases, Atherosclerosis

## Abstract

The inflammatory burden as measured by high-sensitivity C-reactive Protein (hsCRP) is recognized as a cardiovascular risk factor, which can however be affected by lifestyle-related risk factors (LRF). Up-to-date the interplay between hsCRP, LRF and presence and extent of atherosclerotic disease is still largely unknown, which we therefore sought to investigate in a contemporary population-based cohort. We included participants from the cross-sectional population-based Hamburg City Health Study. Affected vascular beds were defined as coronary, peripheral, and cerebrovascular arteries. LRF considered were lack of physical activity, overweight, active smoking and poor adherence to a Mediterranean diet. We computed multivariable analyses with hsCRP as the dependent variable and LRF as covariates according to the number of vascular beds affected. In the 6765 individuals available for analysis, we found a stepwise increase of hsCRP concentration both according to the number of LRF present as well as the number of vascular beds affected. Adjusted regression analyses showed an independent association between increasing numbers of LRF with hsCRP levels across the extent of atherosclerosis. We demonstrate increasing hsCRP concentrations according to both the number of LRF as well as the extent of atherosclerosis, emphasizing the necessity of lifestyle-related risk factor optimization.

## Introduction

Inflammation is considered to be a residual cardiovascular risk factor in patients with cardiovascular disease, even after optimization of other risk factors such as lipid levels^1^. Recently, the Canakinumab Antiinflammatory Thrombosis Outcome Study (CANTOS), the Colchicine Cardiovascular Outcomes Trial (COLCOT) and the Low-Dose Colchicine 2 (LoDoCo2) trials have significantly advanced our understanding of the causal relationship between inflammation and the development and progression of atherosclerosis. Here, an improved outcome in patients with known atherosclerotic cardiovascular disease (ASCVD) was documented after therapeutic targeting of essential steps within the inflammatory cascade^2–4^.

High-sensitivity C-reactive Protein (hsCRP) has become the mainstay in the quantification of the inflammatory burden and has been shown to associate with cardiovascular outcome both in the general population and patients with ASCVD^5–7^. Within the broad spectrum of atherosclerosis, manifestations include coronary artery disease (CAD), cerebrovascular disease (CeVD) and lower extremity peripheral artery disease (PAD).

Interestingly, levels of markers of systemic inflammation correlate with the number of vascular beds affected by atherosclerosis^8^. Moreover, atherosclerosis itself can influence further inflammatory processes^9^. In addition, hsCRP is influenced by numerous factors such as lack of physical activity, poor diet, smoking and elevated body-mass-index (BMI)^10–13^. These factors have been termed lifestyle-related risk factors (LRF). Optimization of LRF via lifestyle changes is associated with a lower inflammatory burden in patients with CAD^14^. Previous studies have considered only a small spectrum of factors influencing hsCRP and, to the best of our knowledge, no previous studies have investigated the impact of these confounders on hsCRP according to the presence and extent of ASCVD.

In the current study, we aim to delineate the association of the inflammatory burden with LRF stratified by the presence and extent of atherosclerosis in a well-defined contemporary population-based cohort.

## Results

### Baseline characteristics

Of the 6765 individuals at baseline 3454 (51.1%) were female and the median age was 61 (Quartile [Q] 1/3: 54, 68) years. Overall, 3480 (51.4%) individuals had no ASCVD, whilst 2418 (35.7%) and 867 (12.8%) participants displayed atherosclerosis in 1 or ≥ 2 vascular beds, respectively. Concerning the individual affected vascular beds, CAD was present in 456 (6.7%) individuals at baseline, whilst CeVD or PAD was documented in 2425 (35.8%) and 1386 (20.5%) of the total study population, respectively. In general, participants with ASCVD and with more extensive ASCVD (i.e. ≥ 2 vascular beds affected) were older and more often male. In addition, co-morbidities such as arterial hypertension, diabetes mellitus and CKD were more likely to be present in individuals with, than those without ASCVD. LDL-c concentrations were comparable in persons without ASCVD (LDL-c: 122.0 [Q1/3: 99.6, 146.1] mg/dl) and those with 1 affected vascular system (LDL-c: 121.3 [Q1/3: 97.2, 145.8] mg/dl), whilst individuals with ≥ 2 vascular beds affected had the lowest registered LDL-c (LDL-c: 106.8 [Q1/3: 79.0, 136.8] mg/dl). Use of statins was higher in participants with 1 (n = 458 [19.0%]) and ≥ 2 (n = 401 [46.2%]) vascular beds compared to those without (n = 269 [7.7%]) ASCVD. Further baseline characteristics are displayed in Table 1, whilst baseline data without multiple imputations are shown in the supplement (Table S4).

**Table 1:** Baseline characteristics of the total study population and according to the number of affected vascular systems.

	Overall (n = 6765)	No atherosclerosis (n = 3480)	1 affected vascular system (n = 2418)	≥ 2 affected vascular systems (n = 867)	*p* value
Age (years)	61.0 (54.0, 68.0)	58.0 (52.0, 64.9)	64.0 (57.0, 70.0)	68.3 (62.6, 72.9)	< 0.0001
Female sex no. (%)	3454 (51.1)	1991 (57.2)	1135 (47.0)	328 (37.8)	< 0.0001
Comorbidities					
Arterial hypertension no. (%)	4095 (63.6)	1735 (53.1)	1632 (70.1)	728 (86.2)	< 0.0001
Diabetes mellitus no. (%)	506 (7.5)	145 (4.2)	208 (8.6)	153 (17.7)	< 0.0001
History of stroke no. (%)	186 (2.8)	0 (0)	96 (4.0)	90 (10.4)	< 0.0001
Chronic kidney disease no. (%)	129 (2.1)	31 (1.0)	55 (2.4)	44 (5.4)	< 0.0001
Medication					
Statins no. (%)	1128 (16.7)	269 (7.7)	458 (19.0)	401 (46.2)	< 0.0001
Antihypertensive medication no. (%)	2323 (36.0)	816 (24.8)	954 (41.2)	553 (65.7)	< 0.0001
Laboratory values					
hsCRP (mg/l)	1.1 (0.6, 2.3)	1.0 (0.5, 2.1)	1.2 (0.6, 2.4)	1.3 (0.7, 2.9)	< 0.0001
Total cholesterol (mg/dl)	208.0 (182.0, 236.0)	210.0 (186.0, 237.6)	208.2 (182.1, 237.1)	192.2 (159.5, 227.6)	< 0.0001
Triglycerides (mg/dl)	98.0 (73.0, 140.0)	93.8 (69.9, 131.3)	101.0 (75.3, 145.4)	113.2 (81.5, 156.5)	< 0.0001
HDL-C (mg/dl)	62.0 (50.0, 76.0)	64.0 (52.1, 78.0)	61.1 (49.7, 75.8)	55.8 (45.4, 69.6)	< 0.0001
LDL-C (mg/dl)	120.0 (96.0, 145.0)	122.0 (99.6, 146.1)	121.3 (97.2, 145.8)	106.8 (79.0, 136.8)	< 0.0001
HbA1C (%)	5.5 (5.3, 5.8)	5.5 (5.3, 5.7)	5.6 (5.3, 5.8)	5.7 (5.4, 6.0)	< 0.0001
Affected vascular systems					
CAD no. (%)	456 (6.7)	-	109 (4.5)	347 (40.1)	< 0.0001
CeVD no. (%)	2425 (35.8)	-	1600 (66.2)	826 (95.3)	< 0.0001
PAD no. (%)	1386 (20.5)	-	710 (29.4)	676 (78.0)	< 0.0001

Missing data of the variables needed for regression analysis and for the classification of subgroups were handled through multivariate imputation by chained equations (MICE). Categorical variables are shown as absolute numbers and percentages, comparison between subgroups was made using the Chi-squared test. Continuous variables are described by median and the 1st/3rd quartile, comparison between subgroups was made using the Kruskall-Wallis test. *CAD* coronary artery disease; *CeVD* Cerebrovascular disease; *HbA1C* Glycated haemoglobin; *HDL-C* high-density lipoprotein cholesterol; *hsCRP* high-sensitivity C-reactive protein; *LDL-C* low-density lipoprotein cholesterol; *PAD* Peripheral artery disease.

Concerning LRF, a total of 4078 (60.3%) persons from the overall cohort were defined as overweight, and 2475 (36.6%) participants had low levels of PA. Poor adherence to a Mediterranean diet was present in 3471 (51.3%) participants, and 1399 (20.7%) were current smokers. Individuals with ≥ 2 vascular beds affected exhibited a higher prevalence of 3 (21.4%) or 4 (5.0%) LRF compared to participants with 1 affected vascular system (3 LRF: 17.3% and 4 LRF: 3.4%) or no atherosclerosis (3 LRF: 14.5% and 4 LRF: 2.4%). Further information concerning LRF is shown in Table 2 and in the supplement (Table S5, without multiple imputations).

**Table 2:** Lifestyle-related risk factors of the total study population and according to the number of affected vascular systems.

	Overall (n = 6765)	No atherosclerosis (n = 3480)	1 affected vascular system (n = 2418)	≥ 2 affected vascular systems (n = 867)	*p* value
LRF					
BMI (kg/m^2^)	26.0 (23.5, 29.0)	25.4 (23.1, 28.4)	26.3 (23.8, 29.3)	27.3 (24.7, 30.3)	< 0.0001
Overweight (BMI ≥ 25 [kg/m^2^])	4078 (60.3)	1913 (55.0)	1535 (63.5)	630 (72.7)	< 0.0001
Obesity (BMI ≥ 30 [kg/m^2^])	1292 (19.1)	549 (15.8)	509 (21.0)	234 (27.0)	< 0.0001
Weekly physical activity (h/week)	2.0 (0, 4.0)	2.0 (0.5, 4.0)	2.0 (0, 4.0)	2.0 (0, 4.0)	0.078
Physical activity (< 1,5 h/week)	2475 (36.6)	1191 (34.2)	916 (37.9)	368 (42.5)	0.0005
sMDS	2.0 (2.0, 3.0)	2.3 (2.0, 3.0)	2.0 (1.9, 3.0)	2.0 (1.2, 3.0)	0.088
sMDS ≤ 2	3471 (51.3)	1755 (50.4)	1248 (51.6)	467 (53.9)	0.21
Current smoking no. (%)	1399 (20.7)	658 (18.9)	524 (21.7)	218 (25.1)	0.0003
Number of LRF					
0 no. (%)	905 (13.4)	552 (15.9)	283 (11.7)	69 (8.0)	< 0.0001
1 no. (%)	2255 (33.3)	1198 (34.4)	802 (33.2)	256 (29.5)	0.085
2 no. (%)	2290 (33.8)	1143 (32.8)	833 (34.5)	313 (36.1)	0.29
3 no. (%)	1108 (16.4)	505 (14.5)	417 (17.3)	186 (21.4)	< 0.0001
4 no. (%)	208 (3.1)	82 (2.4)	82 (3.4)	43 (5.0)	0.0019

Missing data of the variables needed for regression analysis and for the classification of subgroups were handled through multivariate imputation by chained equations (MICE). Categorical variables are shown as absolute numbers and percentages, comparison between subgroups was made using the Chi-squared test. Continuous variables are described by median and the 1st/3rd quartile, comparison between subgroups was made using the Kruskall-Wallis test. BMI body mass index; LRF lifestyle-related risk factors; sMDS simple Mediterranean diet score.

### Distribution of hsCRP according to LRF and number of vascular beds affected

**Figure 1:**
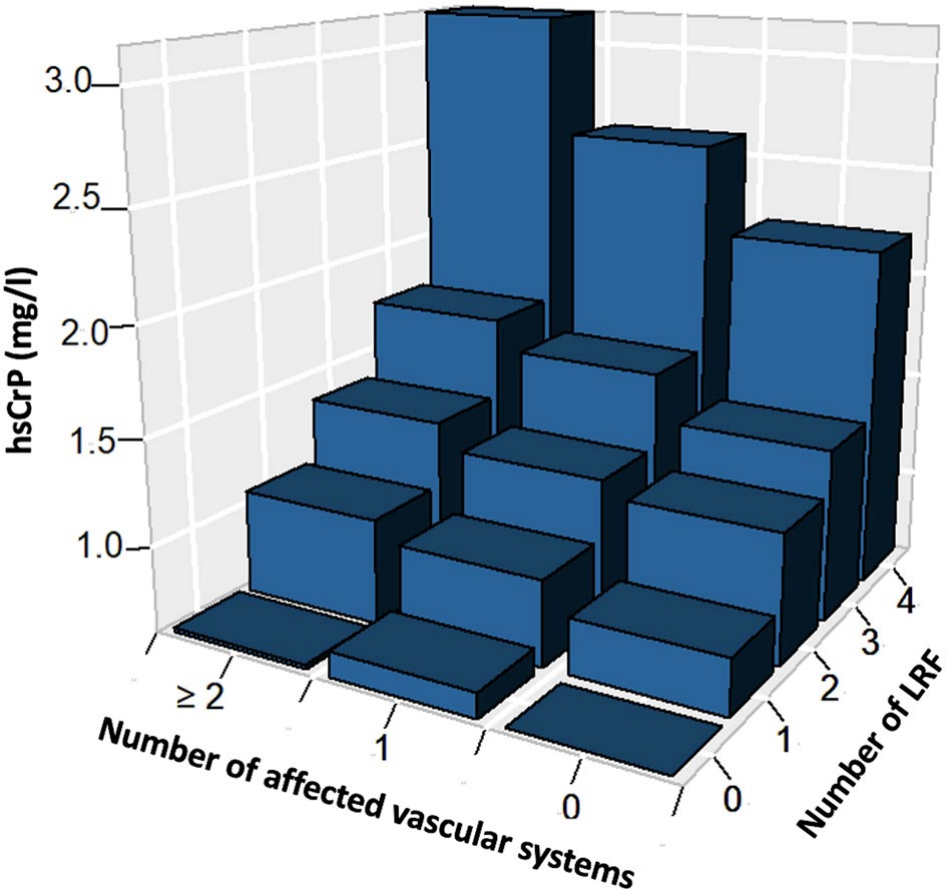
Median high-sensitivity C-reactive protein levels according to the number of affected vascular systems and number of lifestyle-related risk factors. *HsCRP* High-sensitivity C-reactive protein; *LRF* Lifestyle-related risk factors.

In the overall cohort, a median hsCRP of 1.1 (Q1/3: 0.6, 2.3) mg/l was documented (*see* Table S2*for sex-specific hsCRP concentrations in the overall cohort)*. We found a stepwise increase of hsCRP levels according to the number of vascular beds affected (hsCRP for no vascular beds affected: 1.0 [0.5, 2.1] mg/l; 1 vascular bed affected: 1.2 [Q1/3: 0.6, 2.4] mg/l; ≥ 2 vascular beds affected: 1.3 [Q1/3: 0.7, 2.9] mg/l; *p* < 0.001) and also with each incremental increase of LRF (hsCRP for 0 LRF: 0.7 [Q1/3: 0.4, 1.2] mg/l; 1 LRF: 0.9 [Q1/3: 0.5, 1.9] mg/l; 2 LRF: 1.2 [Q1/3: 0.7, 2.6] mg/l; 3 LRF: 1.5 [Q1/3: 0.8, 3.1] mg/l; 4 LRF: 2.5 [Q1/3: 1.2, 4.4] mg/l; *p* < 0.001). When stratifying by number of affected vascular systems and number of LRF, individuals with ≥ 2 vascular beds affected and 4 LRF had the highest concentrations of hsCRP (hsCRP: 3.22 [Q1/3: 1.57, 5.35] mg/l) in comparison to those without ASCVD and no LRF (hsCRP: 0.6 [Q1/3: 0.38, 1.2] mg/l; *see* Fig. 1*and* Table S6 + Figure S1*without multiple imputations*)

.

### Association of hsCRP with LRF

In unadjusted analysis, the number of LRF showed a strong association with hsCRP both in the total cohort as well as according to the number of affected vascular beds (*see* Table S7). After adjusting for confounders, the association of LRF with hsCRP proved to be consistent in the overall cohort (1 LRF: Beta 0.32 [95% CI 0.23, 0.42], *p* < 0.001; 2 LRF: Beta 0.59 [95% CI 0.50, 0.67], *p* < 0.001; 3 LRF: Beta 0.76 [95% CI 0.66, 0.85], *p* < 0.001; 4 LRF: Beta 1.17 [95% CI 1.01, 1.35], *p* < 0.001), and across all groups of ASCVD extent (*see* Table 3*, and furthermore* Table S8*for analyses without multiple imputations*).

**Table 3:** Fully adjusted linear regression analysis for the association of LRF with hsCRP according to the extent of ASCVD.

Overall		
Number of LRF	Beta (95% CI)	*p* value
1	0.32 (0.23, 0.42)	< 0.001
2	0.59 (0.50, 0.67)	< 0.001
3	0.76 (0.66, 0.85)	< 0.001
4	1.17 (1.01, 1.35)	< 0.001

No atherosclerosis		
Number of LRF	Beta (95% CI)	*p* value
1	0.30 (0.18, 0.42)	< 0.001
2	0.59 (0.48, 0.70)	< 0.001
3	0.70 (0.57, 0.83)	< 0.001
4	1.05 (0.79, 1.31)	< 0.001

1 affected vascular system		
Number of LRF	Beta (95% CI)	*p* value
1	0.33 (0.16, 0.49)	< 0.001
2	0.55 (0.40, 0.71)	< 0.001
3	0.78 (0.60, 0.95)	< 0.001
4	1.19 (0.90, 1.47)	< 0.001

≥ 2 affected vascular systems		
Number of LRF	Beta (95% CI)	*p* value
1	0.45 (0.13, 0.77)	0.007
2	0.66 (0.36, 0.97)	< 0.001
3	0.87 (0.56, 1.18)	< 0.001
4	1.29 (0.85, 1.74)	< 0.001

Missing data of the variables needed for regression analysis and for the classification of subgroups were handled through multivariate imputation by chained equations (MICE). The regression coefficient (Beta) and the 95% confidence interval (95% CI) are given. HsCRP was log-transformed. Adjustment was made for age, sex, diabetes, arterial hypertension, intake of statins and chronic kidney disease. The category with no LRF served as reference. *ASCVD* atherosclerotic cardiovascular disease; *hsCRP* high-sensitivity C-reactive protein; *LRF* lifestyle-related risk factors.

Regarding the relation of hsCRP with each individual LRF, overweight consistently displayed the strongest association with hsCRP across the extent of ASCVD (total cohort: Beta 0.68 [95% CI 0.63, 0.73], participants without atherosclerosis: Beta 0.69 [95% CI 0.61, 0.76], participants with 1 affected vascular system: Beta 0.67 [95% CI 0.58, 0.77], participants with ≥ 2 affected vascular systems: Beta 0.63 [95% CI 0.47, 0.79]; all *p* < 0.001) after multivariable adjustment. Other LRF such as smoking showed a consistent and significant association with hsCRP across the extent of ASCVD, whilst the association of PA, and sMDS with hsCRP varied (*see *Table S9,* and furthermore* Table S10*for analyses without multiple imputations*).

## Discussion

The current study investigating the association of hsCRP with LRF stratified by the presence and extent of ASCVD in this large-scale contemporary population-based cohort yielded the following main findings:

1. An increase in hsCRP concentrations was found with both an increasing number of LRF and with a greater extent of ASCVD.
2. The highest concentration of hsCRP was found in individuals with the greatest atherosclerotic burden and most LRF, whilst the lowest concentrations were documented in patients without ASCVD and LRF.
3. Even after controlling for major confounders a significant and independent relationship between number of LRF and hsCRP levels was noted, with increasing trend across the extent of ASCVD.

Overall, this underscores the strong association between LRF, low-grade inflammation quantified by hsCRP, and the presence and extent of ASCVD.

Our study represents a contemporary central European population-based cohort with a high rate of ASCVD, since approximately half of the total sample studied presented with atherosclerosis in either the coronary, peripheral or cerebral arteries. With 6.7% the prevalence of CAD in our cohort is comparable to other Western European large scale studies, such as the UK Biobank, where 4.6% of all participants reported atherosclerotic coronary changes^15^. However, a strikingly high rate of PAD with 20.5% and CeVD in 35.8% of the cohort was determined. This is in concurrence with a previously published investigation of the overall HCHS cohort (n = 10,000 participants), where PAD and CeVD were diagnosed in a similar frequency (23.6% and 30.2%, respectively)^16^. In our study, CeVD was defined either by medical history or through imaging findings on carotid ultrasound incorporating intima media thickness (IMT), plaques and carotid stenosis. This in contrast to other population-based cohorts such as the Study of Health in Pomerania, where prevalent ASCVD was registered by participant history only (20% of the overall cohort at baseline)^17^. A recent meta-analysis investigating the prevalence of CeVD (investigating IMT and hemodynamically relevant stenosis of the carotid arteries, respectively) among individuals from 30 to 79 years of age is in line with our findings. Here, a carotid stenosis was diagnosed in 1.5% of the study population, whilst in 24.4% of all investigated individuals either a carotid plaque or increased IMT was registered^18^.

With regard to lower extremity PAD prevalence our findings are in agreement with other studies, albeit a varying prevalence has been reported on a population-based level in the literature (e.g. 21% pathological ABI in the German Epidemiological Trial on Ankle Brachial Index study and 17.8% in the Prevalence of Peripheral Arterial Disease in Subjects with a Moderate Risk of Cardiovascular Disease in Primary Prevention population)^19, 20^. In our study both imaging and non-invasive tests such as ABI measurement were used to diagnose PAD. Hence the relatively increased proportions of CeVD and lower extremity PAD are most likely due to the use of non-invasive tests, which identified subclinical atherosclerosis. In summary our study paints a realistic picture of prevalent ASCVD in a European middle-aged population-based sample, underpinning the essential role of screening methods for the early identification of atherosclerotic changes within the arterial vascular tree.

Concerning the overall inflammatory burden in our study, we report low concentrations of hsCRP with a median of 1.1 mg/l in the total study cohort. Our findings are corroborated by reports from other population-based cohorts. Steppuhn and colleagues reported a median hsCRP level of 1.15 mg/l from the German Health Interview and Examination Survey for Adults investigating 7006 adults aged 18 to 79 years^21^. Slightly higher hsCRP concentrations of 1.5 mg/l were present in the European prospective investigation into cancer in Norfolk population study (EPIC)^22^. Moreover, in diseased cohorts such as the INTERCATH study a median hsCRP of 1.8 mg/l was noted in a cohort of 1014 CAD patients^14^. Whilst the inflammatory burden in our overall cohort was low, we were able to demonstrate that both a higher burden of LRF and a greater extent of ASCVD is associated with rising hsCRP concentrations. This independent association of the number of LRF with hsCRP concentrations was seen across the strata of ASCVD. Our findings have both clinical and scientific implications. Recently, Ridker and colleagues were able to demonstrate that in patients already treated with statins the inflammatory burden as measured by hsCRP was the strongest predictor for major adverse cardiovascular events, and both cardiovascular as well as all-cause mortality^7^. In a study from Blaum and colleagues, the investigators were able to demonstrate that the number of LRF was strongly associated with hsCRP concentrations. Furthermore, and similar to our results, the authors demonstrated in their CAD cohort that overweight had the strongest association with the inflammatory burden^14^. This finding has been validated in multiple studies where adiposity was shown to correlate with an inflammatory state, potentially through secretion of pro-inflammatory cytokines such as IL-6 or TNF-a by adipose tissue^13, 23^. Accordingly, a positive effect on the inflammatory load after weight loss has been demonstrated in overweight patients^24, 25^. The anti-inflammatory potential inherent in risk factor optimization is underlined in the study by Blaum and colleagues, where 37.9% of the study cohort would achieve hsCRP levels < 2 mg/l after a hypothesized optimization of risk factors (i.e. weight loss, smoking cessation, adherence to a mediterranean diet, regular PA), and therefore to levels below the threshold used in the enrolment of seminal trials investigating anti-inflammatory treatment (i.e. CANTOS and COLCOT)^3, 4, 14^. Thus, solely by the optimization of their LRF burden, a relevant health benefit could be unlocked by the patients themselves ahead of the initiation of a specific anti-inflammatory therapy and the potentially associated side-effects of these medications. Our findings therefore underline the necessity of lifestyle optimization as recommended in the guidelines on cardiovascular disease prevention in clinical practice from the European Society of Cardiology^26^.

In our cohort individuals with the greatest atherosclerotic burden (i.e. ≥ 2 vascular systems affected) and most LRF had the highest concentrations of hsCRP, displaying the synergistic effect of atherosclerosis itself and LRF on the overall inflammatory burden. Patients with atherosclerosis in two or more vascular beds are a sub-population in whom a particularly high risk for recurrent events has been described^27^. An intensified approach to secondary prevention has therefore been proposed to reduce the incidence of adverse cardiovascular events and mortality in this patient population^28^. Accordingly, optimization of LRF should particularly be prioritized in this cohort.

On the other end of the spectrum, we were also able to show that in participants without ASCVD, the number of LRF was significantly and independently associated with hsCRP concentrations. Besides the use of CRP as biomarker to assess the residual inflammatory risk, the causal role of this acute-phase protein in the development of ASCVD has also been investigated^9^. A contribution of CRP to endothelial dysfunction and hypertension by the inhibition of nitric oxide, impaired endothelial-associated vascular relaxation, and association with plaque instability by activating NF-κB has been demonstrated^29^. Therefore, it can be hypothesized that the optimization of LRF might be associated with a delayed development of atherosclerotic precursors. However, further studies are needed to ascertain these findings in primary prevention.

## Limitations

Whilst the studied population represents a large contemporary cohort, some limitations merit consideration. Since random recruitment took place from a statistical sample of a middle-aged population of the German city of Hamburg, a translation of our findings to other ethnicities, age groups, or geographical regions can only be carried out with caution. Moreover, whilst the selection of participants was carried out randomly, a recruitment bias is possible since healthier individuals are more likely to accept taking part in a study. Also, a relevant proportion of participants (32%) of the overall cohort of 10,000 individuals had to be excluded due to missing hsCRP samples, and comorbidities such as prevalent neoplastic diseases or inflammatory disorders. Lastly, whilst CeVD and PAD were deemed present either by medical history or imaging studies, for CAD solely self-reported information from the individual participants’ medical history was available. However, the CAD prevalence reported in our study is comparable to other large-scale population-based cohorts such as the UK Biobank.

## Conclusion

In this contemporary population-based cohort, a significant association of lifestyle-related risk factors such as diet, physical activity, smoking, and overweight with the inflammatory burden as measured by hsCRP across the extent of atherosclerotic disease was determined. We demonstrate that both the presence and extent of ACSVD and the burden of LRF have additive effects on the inflammatory load. These findings emphasize the important role of subclinical inflammation in individuals with and without ASCVD and might be helpful for the definition of target populations for anti-inflammatory compounds across the extent of atherosclerotic disease.

## Methods

### Study cohort

The Hamburg City Health Study (HCHS) is a prospective population-based cohort study that aims to gather extensive data on risk factors for chronic diseases, with a focus on organ system function and structure through a wide range of assessments. It involves 45,000 randomly selected participants from Hamburg, Germany, aged 45 to 74, and includes a detailed examination of lifestyle, environment, genetics, and health outcomes. This large-scale, long-term assessment, conducted in a European metropolitan population, seeks to explore the interplay between biological and psychosocial factors in the context of health and disease. Hereby, the HCHS aims to identify risk factors for major chronic diseases and survivorship, with the goal of accurately determining the prevalence and incidence of common diseases, and the development of complex models for predicting health outcomes. More details on the study are described elsewhere^30^.

The current cross-sectional analysis incorporates data from the first 10,000 individuals. Baseline examinations, which included the completion of validated patient questionnaires to self-report lifestyle, blood draws, and non-invasive tests, were conducted during a single-day visit at the HCHS Epidemiological Study Center of the University Medical Center Hamburg-Eppendorf between 2016 and 2018. An ethics approval was obtained from the ethics committee of the Medical Association of Hamburg (PV5131) and the study is registered at ClinicalTrials.gov (NCT03934957). Written informed consent was obtained from all participants. Moreover, the study and all applied methods were performed in accordance with the relevant guidelines and regulations.

### In- and exclusion criteria

**Figure 2:**
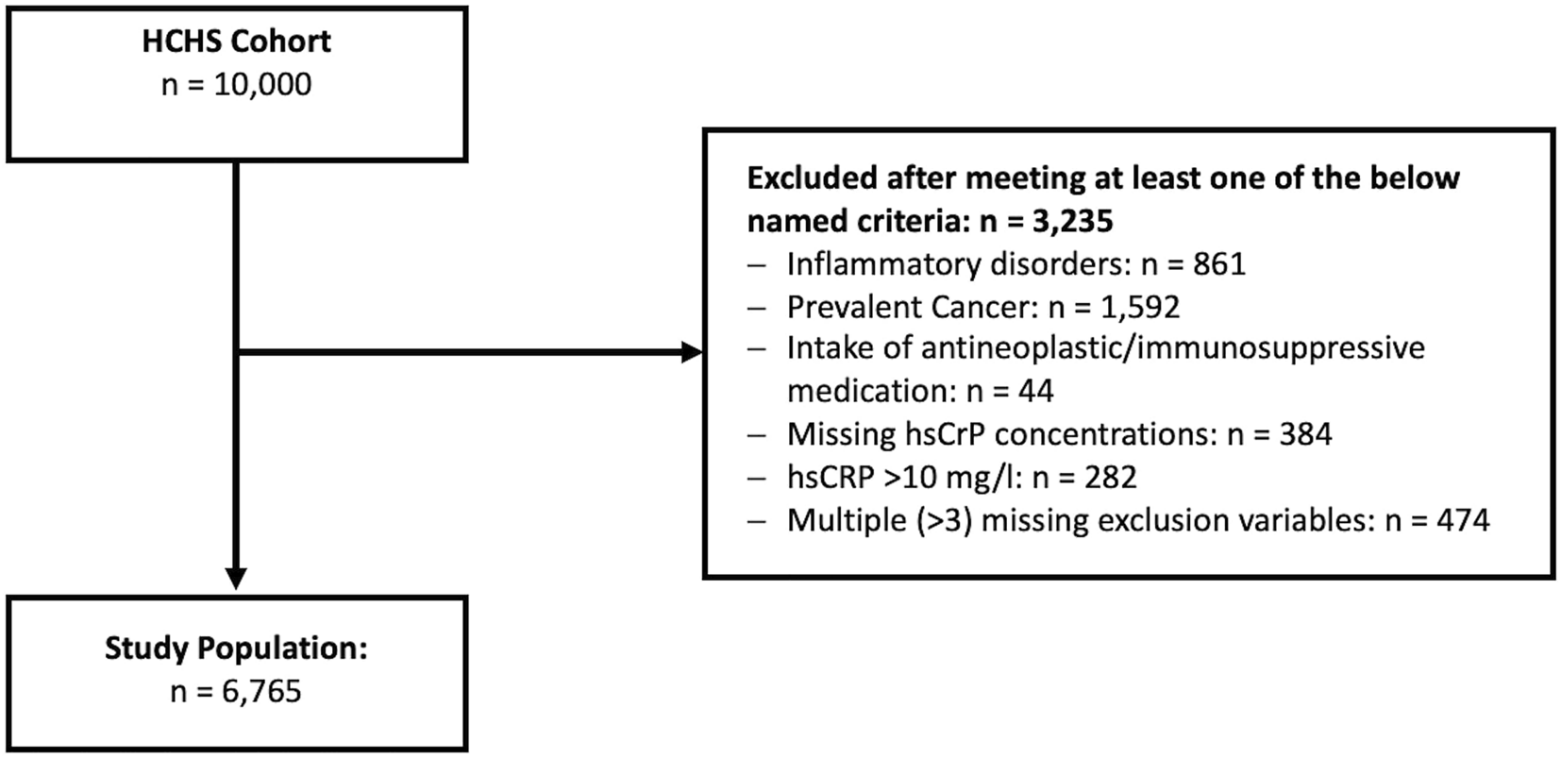
Study flowchart. *HCHS* Hamburg city health study; *hsCRP* High-sensitivity C-reactive protein.

Participants with valid measurements of hsCRP (i.e. within the limit of detection of the used assay) concentration at baseline were included in the current study. Individuals with chronic inflammatory disorders (defined as rheumatoid arthritis, multiple sclerosis, psoriasis, inflammatory bowel disease), prevalent cancer, intake of immunosuppressive or antineoplastic medication, or missing information of more than 3 values of the previously mentioned criteria were excluded. Also, participants with missing hsCRP concentrations or hsCRP concentrations > 10 mg/l compatible with an acute infection were excluded. After applying the in- and exclusion criteria 6675 datasets were left for the presented analyses (see Fig. 2 for further details). The reporting of the current study was in accordance with the Strengthening the reporting of observational studies in epidemiology (STROBE) statement^31^.

### Assessment of cardiovascular risk factors, co-morbidities and further variables

Intake of medications was validated via Anatomical Therapeutic Chemical Classification System Codes (ATC-Code) for anti-hypertensive, anti-diabetic medications and statins. Diabetes mellitus was defined as taking medication of the ATC group A10, a fasting glucose > 126 mg/dL, a non-fasting glucose > 200 mg/dL, an HbA1C > 6.5%, or self-reported diabetes. Arterial hypertension was considered to be prevalent when taking medication in ATC group C09A, C09C, C07A, C03C, C03A, C03D, C08C, C02D, C02A, C09X, C01D, a measured resting systolic blood pressure > 140 mmHg/resting diastolic blood pressure > 90 mmHg at inclusion, or self-reported hypertension. Prevalent chronic kidney disease (CKD) was defined as estimated glomerular filtration rate (eGFR) below 60 ml/min as estimated by the CKD-EPI formula or by medical history^32^.

### Laboratory measurements

Laboratory measurements (including quantification of glycated hemoglobin (HbA1C), cholesterol levels and hsCRP) were carried out at the HCHS laboratories on the same day as the baseline examination. HsCRP was quantified using a commonly available assay (SIEMENS Healthineers, Atellica High Sensitivity C-reactive protein; Range: 0.16–10.00 mg/l, limit of detection ≤ 0.16 mg/l). Calibration was carried out within the clinical routine.

### Assessment of atherosclerotic burden

Prevalent CAD was assessed via self-reported medical history. CeVD was diagnosed by medical history including previous stroke, or by IMT of ≥ 1 mm, vascular plaque (defined as localized IMT thickening of ≥ 1.5 mm), or stenoses (defined as systolic flow velocity > 200 m/s in the common, internal and external carotid artery) on carotid ultrasound at inclusion^33^. PAD was diagnosed via medical history, or pathological ankle-brachial-index (ABI,  ≤ 0.9)^34^. Proportions of patients with history of ASCVD and atherosclerosis detected at baseline (for CeVD and PAD) are shown in Table S1.

### Assessment of lifestyle-related risk factors

LRF that were taken into consideration were lack of physical activity (PA), defined as < 1.5 h/week of exercise, overweight, defined as BMI ≥ 25 kg/m^2^, current smoking, defined as active smoking or recently quitted smoking within the last 6 months, and poor adherence to a Mediterranean diet. A simple Mediterranean diet score (sMDS) as used in the Stabilisation of atherosclerotic plaque by initiation of darapladib therapy (STABILITY) trial was calculated to investigate dietary habits^35^. Briefly, participants answered a food questionnaire in which 4 food groups (consumption of fruit, vegetables, fish, and alcohol) were queried, and points according to the frequency of consumption (maximum 2 points, minimum 0 points) were distributed (see supplementary Table S3). A score ranging from 0 to 8 points was then calculated. Poor adherence to Mediterranean diet was defined as an sMDS ≤ 2 points.

### Statistical analyses

Categorical variables are shown as absolute numbers and percentages, compared using Chi-squared test. Continuous variables are reported as median and 1st/3rd quartile (Q1/3) and were compared using Kruskall-Wallis test. To analyse the burden of ASCVD, the population was divided into subgroups according to the number of vascular beds affected (no ASCVD, 1 affected vascular system,  ≥ 2 affected vascular systems). Uni- and multivariable linear regression models in the overall cohort and each subgroup with logarithmic hsCRP as dependent variable and LRF (both number of LRF as a categorical variable and each individual LRF) as covariate were calculated and adjusted for age, sex, diabetes, arterial hypertension, intake of statins and chronic kidney disease. Missing data of the variables needed for regression analysis and for the classification of subgroups (overweight, PA, sMDS, smoking, diabetes, CAD, PAD and CeVD) were handled by Multivariate Imputation by Chained Equations (MICE), as proposed by Buuren and Groothuis-Oudshoorn (20 imputed data sets, R Package MICE)^36^. Results were considered as statistically significant at a significance level of a two-sided alpha < 0.05. All statistical tests were carried out using R version 4.1.2.

### Previous Presentation

Part of this work has been presented as an abstract presentation at the Congress of the European Society of Cardiology 2023.

### Supplementary Information

Supplementary Information.

## Data Availability

The data underlying this article cannot be shared publicly due to the privacy of individuals that participated in the study. The data will be shared on reasonable request to the corresponding author.
