# Publisher Correction: Inflammatory burden, lifestyle and atherosclerotic cardiovascular disease: insights from a population based cohort study

**DOI:** 10.1038/s41598-024-51444-6

**Published:** 2024-01-08

**Authors:** Benjamin Bay, Christopher Blaum, Caroline Kellner, Ramona Bei der Kellen, Francisco Ojeda, Julia Waibel, Natalie Arnold, Christian‑A. Behrendt, David L. Rimmele, Goetz Thomalla, Raphael Twerenbold, Stefan Blankenberg, Birgit Zyriax, Fabian J. Brunner, Christoph Waldeyer

**Affiliations:** 1 Department of Cardiology, University Heart and Vascular Center Hamburg, University Medical Center Hamburg-Eppendorf, Martinistrasse 52, 20246 Hamburg, Germany; 2 German Center for Cardiovascular Research (DZHK), Partner Site Hamburg/Kiel/Lübeck, Hamburg, Germany; 3 Center for Population Health Innovation (POINT), University Heart and Vascular Center Hamburg, University Medical Center Hamburg-Eppendorf, Hamburg, Germany; 4 Department of Neurology, University Medical Center Hamburg-Eppendorf, Hamburg, Germany; 5 Research Group Preventive Medicine and Nutrition, Midwifery Science‑Health Care Research and Prevention (IVDP), University Medical Center Hamburg-Eppendorf, Hamburg, Germany

Correction to: *Scientific Reports* 10.1038/s41598-023-48602-7, published online 08 December 2023

The Funding section in the original version of this Article was incomplete.

“Open Access funding enabled and organized by Projekt DEAL.”

now reads:

"Open Access funding enabled and organized by Projekt DEAL. We acknowledge financial support from the Open Access Publication Fund of UKE—Universitätsklinikum Hamburg-Eppendorf and DFG—German Research Foundation. The HCHS is supported by the Innovative medicine initiative (Grant number 116074), by the Joachim Herz Foundation, by the Foundation Leducq (Grant number 16 CVD 03), by the euCanSHare grant agreement (Grant number 825903-euCanSHare H2020), and the Deutsche Forschungsgemeinschaft (Grant number TH1106/5-1; AA93/2-1). Furthermore, it is supported by the participating institutes and departments from the University Medical Centre Hamburg-Eppendorf, which contribute with individual and scaled budgets to the overall funding. Technical equipment is provided by SIEMENS according to a contract for 12 years, the Schiller AG on a loan basis for six years, and Topcon on a loan basis from 2017 until 2022. The Hamburg City Health Study is additionally supported by an unrestricted grant (2017 to 2022) by Bayer. Project-related analyses are supported by Amgen, Astra Zeneca, BASF, Deutsche Gesetzliche Unfallversicherung (DGUV), Deutsches Krebsforschungszentrum (DKFZ), Deutsches Zentrum für Herz-Kreislauf-Forschung (DZHK), Deutsche Stiftung für Herzforschung, Novartis, Seefried Stiftung, and Unilever. The study is further supported by donations from the “Förderverein zur Förderung der HCHS e.V.”, TePe® (2014) and Boston Scientific (2016). A current list of the supporters is online available on www.uke.de/hchs. Sponsor funding has in no way influenced the content or management of this study.”

The original Article has been corrected.

