# Supplementary material for: Inflammatory burden, lifestyle and atherosclerotic cardiovascular disease: insights from a population based cohort study
Source: Sci Rep. 2023 Dec 8;13:21761. doi: 10.1038/s41598-023-48602-7 (PMC10709308; doi:10.1038/s41598-023-48602-7)
Supplement: Supplementary file 1 — Supplementary Information. [file 41598_2023_48602_MOESM1_ESM.docx]

**
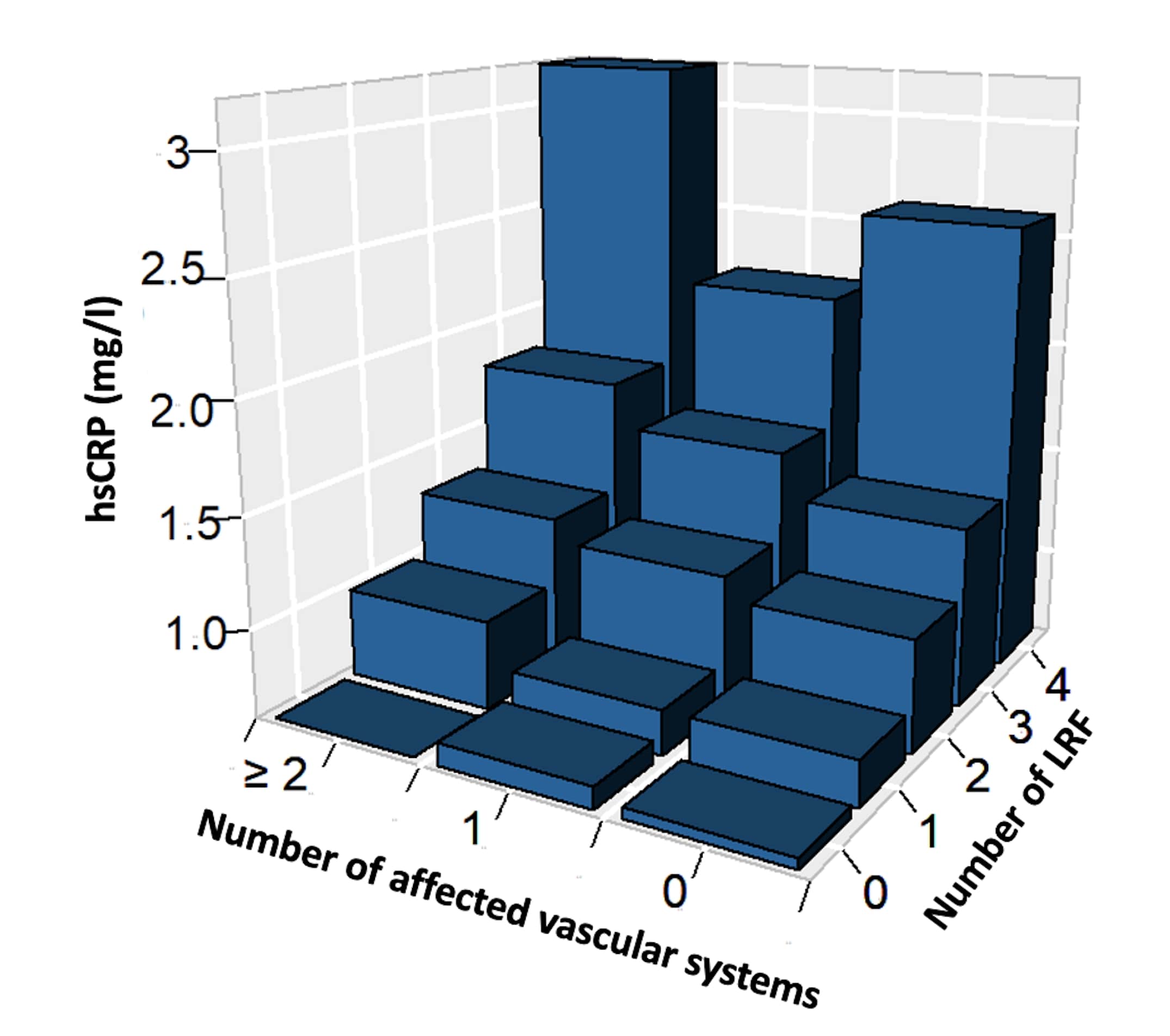
**

**Figure S1:** **HsCRP levels according to the number of affected vascular systems and number of LRF without multiple imputation.** *HsCRP* high-sensitivity C-reactive protein; *LRF* lifestyle-related risk factors

|  | **CAD** | **CeVD** | **PAD** |
| --- | --- | --- | --- |
| Patient History^#^ No. (%) | 456 (100) | 437 (18.0) | 251 (18.1) |
| Diagnosed during baseline examination* No. (%) | - | 1,988 (82.0) | 1,135 (81.9) |
| Overall No. (%) | 456 (100) | 2,425 (100) | 1,386 (100) |

**Table S1: Proportions of Coronary artery disease, Cerebrovascular disease and Peripheral artery disease diagnosed by patient history and non-invasive tests at baseline.** Variables are shown as absolute numbers and percentages. *CAD* coronary artery disease; *CeVD* Cerebrovascular disease; *PAD* Peripheral artery disease. ^#^Patient history was ascertained using a patient questionnaire. *Non-invasive tests were carried out at baseline for PAD (Ankle brachial index) and CeVD (Carotid ultrasound), but not for CAD.

|  | **All (N=6,756)** | **Male (N=3,311)** | **Female (N=3,454)** | **p-value** |
| --- | --- | --- | --- | --- |
| hsCRP (mg/l) | 1.1 (0.6, 2.3) | 1.1 (0.6, 2.2) | 1.1 (0.6, 2.4) | 0.75 |

**Table S2: HsCRP concentrations according to sex.** High-sensitivity C-reactive protein concentrations are displayed as median and the 1st/3rd quartile. *HsCRP* high-sensitivity C-reactive protein

| **Food** | **0 points** | **1 point** | **2 points** |
| --- | --- | --- | --- |
| Fruit | <1-2 every day | 1-2 serves /day | 3+ serves / day |
| Vegetables | <1-2 every day | 1-2 serves/day | 3+ serves /day |
| Fish | <1-2 every week | 1/week | ≥2/ week |
| Alcohol | None or potentially hazardous | Women 1-3 drinks/week  Men 1-5 drinks/week | Women ≥4 drinks /week  Men ≥6 drinks /week  and no hazardous drinking |

**Table S3: Calculation of the simple Mediterranean Diet Score (sMDS).** Potentially hazardous drinking was defined as >6 drinks/day at one time at least once/week.

|  | **Overall**  **(n = 3,347)** | **No atherosclerosis**  **(n = 1,591)** | **1 affected vascular system**  **(n = 1,134)** | **≥2 affected vascular systems**  **(n = 622)** | **p-value** |
| --- | --- | --- | --- | --- | --- |
| Age (years) | 62.0 (54.5, 69.0) | 58.0 (52.0, 64.0) | 64.0 (56.0, 70.0) | 69.0 (63.0, 73.0) | <0.0001 |
| Female sex No. (%) | 1,617 (48.3) | 892 (56.1) | 510 (45.0) | 215 (34.6) | <0.0001 |
| **Comorbidities** |  |  |  |  |  |
| Arterial Hypertension No. (%) | 1,986 (61.6) | 715 (47.3) | 736 (66.7) | 535 (88.0) | <0.0001 |
| Diabetes mellitus No. (%) | 261 (7.9) | 52 (3.3) | 94 (8.4) | 115 (18.7) | <0.0001 |
| History of Stroke No. (%) | 112 (3.4) | - | 43 (3.8) | 69 (11.4) | <0.0001 |
| Chronic Kidney Disease No. (%) | 60 (1.9) | 7 (0.5) | 20 (1.9) | 33 (5.7) | <0.0001 |
| **Medication** |  |  |  |  |  |
| Statins No. (%) | 610 (19.0) | 105 (6.9) | 184 (16.8 | 321 (52.8) | <0.0001 |
| Antihypertensive medication No. (%) | 1,151 (35.8) | 320 (21.2) | 406 (37.2) | 425 (69.9) | <0.0001 |
| **Laboratory values** |  |  |  |  |  |
| hsCRP (mg/l) | 1.1 (0.6, 2.2) | 1.0 (0.5, 1.9) | 1.1 (0.6, 2.4) | 1.3 (0.7, 2.8) | <0.0001 |
| Total cholesterol (mg/dl) | 205.0 (178.0, 233.0) | 209.0 (185.0, 235.0 | 207.0 (181.0, 235.0) | 186.0 (154.0, 222.0) | <0.0001 |
| Triglycerides (mg/dl) | 96.0 (72.0, 136.0) | 91.0 (68.0, 124.0) | 95.0 (73.0, 139.0) | 114.0 (82.2, 156.8) | <0.0001 |
| HDL-c (mg/dl) | 61.0 (50.0, 75.0) | 64.0 (52.0, 77.0) | 62.0 (49.0, 76.0) | 54.0 (44.0, 68.0) | <0.0001 |
| LDL-c (mg/dl) | 119.0 (93.0, 142.0) | 123.0 (99.0, 144.0) | 121.0 (97.0, 143.0) | 102.0 (75.0, 133.2) | <0.0001 |
| HbA1C (%) | 5.5 (5.3, 5.8) | 5.4 (5.2, 5.7) | 5.5 (5.3, 5.8) | 5.7 (5.5, 6.1) | <0.0001 |
| **Affected vascular systems** |  |  |  |  |  |
| CAD No. (%) | 361 (10.9) | - | 43 (3.8) | 318 (53.1) | <0.0001 |
| CeVD No. (%) | 1,335 (40.1) | - | 746 (65.8) | 589 (97.0) | <0.0001 |
| PAD No. (%) | 61 (23.6) | - | 345 (30.4) | 416 (83.5) | <0.0001 |

**Table S4: Baseline characteristics of the total study population and according to the number of affected vascular systems without multiple imputations**. Categorical variables are shown as absolute numbers and percentages, comparison between subgroups was made using the Chi-squared test. Continuous variables are described by median and the 1st/3rd quartile, comparison between subgroups was made using the Kruskall-Wallis test. *CAD* coronary artery disease; *CeVD* Cerebrovascular disease; *HbA1C* Glycated hemoglobin; *HDL-c* high-density lipoprotein cholesterol; *hsCRP* high-sensitivity C-reactive protein; *LDL-c* low-density lipoprotein cholesterol; *PAD* Peripheral artery disease.

|  | **Overall**  **(n = 3,347)** | **No atherosclerosis**  **(n = 1,591)** | **1 affected vascular system**  **(n = 1,134)** | **≥2 affected vascular systems**  **(n = 622)** | **p-value** |
| --- | --- | --- | --- | --- | --- |
| **LRF** |  |  |  |  |  |
| BMI (kg/m2) | 25.8 (23.4, 28.8) | 25.1 (23.0, 28.0) | 25.9 (23.6, 29.1) | 27.3 (24.9, 30.3) | <0.0001 |
| BMI **≥**25 (kg/m2) | 1,881 (58.9) | 789 (51.9) | 651 (60.3) | 441 (74.1) | <0.0001 |
| BMI **≥**30 (kg/m2) | 584 (18.3) | 219 (14.4) | 202 (18.7) | 163 (27.4) | <0.0001 |
| Physical activity (h/week) | 2.0 (0, 4.0) | 2.0 (0.1, 4.0) | 2.0 (0, 4.0) | 1.5 (0, 4.0) | 0.0002 |
| Weekly physical activity (h/week) | 1,200 (39.5) | 523 (36.0) | 414 (39.7) | 263 (48.5) | <0.0001 |
| sMDS | 2.0 (2.0, 3.0) | 2.0 (2.0, 3.0) | 2.0 (2.0, 3.0) | 2.0 (2.0, 3.0) | 0.33 |
| sMDS <2 | 1,418 (51.8) | 688 (51.5) | 488 (51.5) | 242 (53.2) | 0.80 |
| Smoking No. (%) | 690 (20.7) | 292 (18.4) | 247 (21.8) | 151 (24.4) | 0.0034 |
| **Number of LRF** |  |  |  |  |  |
| - 0 No. (%) | 335 (13.1) | 196 (15.7) | 106 (12.0) | 33 (7.9) | <0.0001 |
| - 1 No. (%) | 794 (31.1) | 398 (31.9) | 287 (32.5) | 109 (26.0) | 0.042 |
| - 2 No. (%) | 847 (33.2) | 417 (33.4) | 293 (33.1) | 137 (32.6) | 0.95 |
| - 3 No. (%) | 481 (18.9) | 206 (16.5) | 162 (18.3) | 113 (26.9) | <0.0001 |
| - 4 No. (%) | 94 (3.7) | 30 (2.4) | 36 (4.1) | 28 (6.7) | 0.0002 |

**Table S5: LRF** **risk factors of the total study population and according to the number of affected vascular systems without multiple imputations**. Categorical variables are shown as absolute numbers and percentages, comparison between subgroups was made using the Chi-squared test. Continuous variables are described by median and the 1st/3rd quartile, comparison between subgroups was made using the Kruskall-Wallis test. *BMI* body mass index; *LRF* lifestyle-related risk factors; *sMDS* simple mediterranean diet score.

|  | **No atherosclerosis** | **1 affected vascular system** | **≥2 affected vascular systems** |
| --- | --- | --- | --- |
| **0 LRF** | 0.6 (0.38, 1.2) | 0.71 (0.4, 1.31) | 0.63 (0.4, 1.23) |
| **1 LRF** | 0.86 (0.49, 1.77) | 1 (0.52, 2.01) | 1.12 (0.59, 2.29) |
| **2 LRF** | 1.2 (0.6, 2.43) | 1.24 (0.68, 2.69) | 1.39 (0.74, 2.89) |
| **3 LRF** | 1.38 (0.76, 2.66) | 1.66 (0.82, 3.47) | 1.8 (0.89, 3.43) |
| **4 LRF** | 2.17 (0.94, 3.69) | 2.54 (1.29, 4.5) | 3.22 (1.57, 5.35) |

**Table S6: Median hsCRP concentrations in mg/l according to the extent of ASCVD and number of LRF with multiple imputation.** Missing data of the variables needed for regression analysis and for the classification of subgroups were handled through multivariate imputation by chained equations (MICE). *ASCVD* atherosclerotic cardiovascular disease; high-sensitivity C-reactive protein concentrations are displayed as median and the 1st/3rd quartile*. LRF* lifestyle-related risk factors.

| **Overall** | | |
| --- | --- | --- |
| **Number of LRF** | **Beta (95% CI)** | **p-value** |
| 1 | 0.35 (0.26, 0.44) | <0.001 |
| 2 | 0.62 (0.54, 0.70) | <0.001 |
| 3 | 0.81 (0.72, 0.90) | <0.001 |
| 4 | 1.19 (1.04, 1.33) | <0.001 |
| **No atherosclerosis** | | |
| **Number of LRF** | **Beta (95% CI)** | **p-value** |
| 1 | 0.32 (0.20, 0.43) | 0.086 |
| 2 | 0.62 (0.52, 0.73) | <0.001 |
| 3 | 0.76 (0.64, 0.88) | <0.001 |
| 4 | 1.08 (0.85, 1.31) | <0.001 |
| **1 affected vascular system** | | |
| **Number of LRF** | **Beta (95% CI)** | **p-value** |
| 1 | 0.33 (0.18, 0.49) | <0.001 |
| 2 | 0.56 (0.41, 0.70) | <0.001 |
| 3 | 0.80 (0.64, 0.97) | <0.001 |
| 4 | 1.14 (0.88, 1.41) | <0.001 |
| **≥2** **affected vascular systems** | | |
| **Number of LRF** | **Beta (95% CI)** | **p-value** |
| 1 | 0.47 (0.17, 0.77) | 0.002 |
| 2 | 0.69 (0.41, 0.98) | <0.001 |
| 3 | 0.87 (0.58, 1.16) | <0.001 |
| 4 | 1.36 (0.95, 1.77) | <0.001 |

**Table S7:** **Unadjusted linear regression analysis for the association of LRF with hsCRP according to the extent of ASCVD with multiple imputation**. Missing data of the variables needed for regression analysis and for the classification of subgroups were handled through multivariate imputation by chained equations (MICE). The regression coefficient (Beta) and the 95% confidence interval (95% CI) are given. *HsCRP* was log-transformed. *ASCVD* atherosclerotic cardiovascular disease; *hsCRP* high-sensitivity C-reactive protein; *LRF* lifestyle-related risk factors.

| **Overall** | | |
| --- | --- | --- |
| **Number of LRF** | **Beta (95% CI)** | **p-value** |
| 1 | 0.28 (0.18, 0.37) | <0.001 |
| 2 | 0.53 (0.43, 0.62) | <0.001 |
| 3 | 0.71 (0.61, 0.82) | <0.001 |
| 4 | 1.16 (0.99, 1.32) | <0.001 |
| **No atherosclerosis** | | |
| **Number of LRF** | **Beta (95% CI)** | **p-value** |
| 1 | 0.16 (0.0, 0.33) | 0.056 |
| 2 | 0.46 (0.29, 0.63) | <0.001 |
| 3 | 0.62 (0.43, 0.81) | <0.001 |
| 4 | 1.05 (0.67, 1.43) | <0.001 |
| **1 affected vascular system** | | |
| **Number of LRF** | **Beta (95% CI)** | **p-value** |
| 1 | 0.18 (-0.04, 0.41) | 0.11 |
| 2 | 0.42 (0.19, 0.65) | <0.001 |
| 3 | 0.77 (0.53, 1.02) | <0.001 |
| 4 | 1.02 (0.64, 1.41) | <0.001 |
| **≥2** **affected vascular systems** | | |
| **Number of LRF** | **Beta (95% CI)** | **p-value** |
| 1 | 0.56 (0.18, 0.94) | 0.004 |
| 2 | 0.86 (0.49, 1.23) | <0.001 |
| 3 | 1.06 (0.68, 1.44) | <0.001 |
| 4 | 1.50 (1.00, 1.99) | <0.001 |

**Table S8:** **Fully adjusted linear regression analysis for the association of LRF** **with hsCRP according to the extent of ASCVD without multiple imputations**. The regression coefficient (Beta) and the 95% confidence interval (95% CI) are given. *HsCRP* was log-transformed. Adjustment was made for age, sex, diabetes, arterial hypertension, intake of statins and chronic kidney disease. *ASCVD* atherosclerotic cardiovascular disease; *hsCRP* high-sensitivity C-reactive protein; *LRF* lifestyle-related risk factors.

| **Overall** | | |
| --- | --- | --- |
| **LRF** | **Beta (95% CI)** | **p-value** |
| Overweight | 0.68 (0.63, 0.73) | <0.001 |
| Physical activity <1.5h/week | 0.10 (0.05, 0.15) | <0.001 |
| sMDS ≤2 | 0.07 (0.02, 0.12) | <0.001 |
| Smoking | 0.25 (0.19, 0.31) | <0.001 |
| **No atherosclerosis** | | |
| **LRF** | **Beta (95% CI)** | **p-value** |
| Overweight | 0.69 (0.61, 0.77) | <0.001 |
| Physical activity <1.5h/week | 0.05 (-0.02, 0.12) | 0.18 |
| sMDS ≤2 | 0.06 (0.0, 0.13) | 0.058 |
| Smoking | 0.19 (0.10, 0.27) | <0.001 |
| **1 affected vascular system** | | |
| **LRF** | **Beta (95% CI)** | **p-value** |
| Overweight | 0.67 (0.58, 0.77) | <0.001 |
| Physical activity <1.5h/week | 0.11 (0.02, 0.20) | 0.02 |
| sMDS ≤2 | 0.08 (0.0, 0.16) | 0.063 |
| Smoking | 0.26 (0.15, 0.37) | <0.001 |
| **≥2** **affected vascular systems** | | |
| **LRF** | **Beta (95% CI)** | **p-value** |
| Overweight | 0.63 (0.47, 0.79) | <0.001 |
| Physical activity <1.5h/week | 0.24 (0.10, 0.39) | 0.001 |
| sMDS ≤2 | 0.03 (-0.13, 0.18) | 0.74 |
| Smoking | 0.33 (0.16, 0.50) | <0.001 |

**Table S9:** **Fully adjusted linear regression analysis for the association of each individual LRF with hsCRP according to the extent of ASCVD with multiple imputation**. Missing data of the variables needed for regression analysis and for the classification of subgroups were handled through multivariate imputation by chained equations (MICE). The regression coefficient (Beta) and the 95% confidence interval (95% CI) are given. *HsCRP* was log-transformed. Adjustment was made for age, sex, diabetes, arterial hypertension, intake of statins and chronic kidney disease. *ASCVD* atherosclerotic cardiovascular disease; *hsCRP* high-sensitivity C-reactive protein; *LRF* lifestyle-related risk factors; *sMDS* simple mediterranean diet score.

| **Overall** | | |
| --- | --- | --- |
| **LRF** | **Beta (95% CI)** | **p-value** |
| Overweight | 0.69 (0.63, 0.75) | <0.001 |
| Physical activity <1.5h/week | 0.09 (0.03, 0.15) | 0.002 |
| sMDS ≤2 | 0.05 (0.0, 0.11) | 0.064 |
| Smoking | 0.26 (0.19, 0.33) | <0.001 |
| **No atherosclerosis** | | |
| **LRF** | **Beta (95% CI)** | **p-value** |
| Overweight | 0.62 (0.51, 0.74) | <0.001 |
| Physical activity <1.5h/week | 0.10 (-0.02, 0.21) | 0.091 |
| sMDS ≤2 | 0.02 (-0.09, 0.12) | 0.77 |
| Smoking | 0.21 (0.07, 0.35) | 0.003 |
| **1 affected vascular system** | | |
| **LRF** | **Beta (95% CI)** | **p-value** |
| Overweight | 0.62 (0.48, 0.76) | <0.001 |
| Physical activity <1.5h/week | 0.13 (0.0, 0.27) | 0.054 |
| sMDS ≤2 | 0.09 (-0.04, 0.22) | 0.18 |
| Smoking | 0.32 (0.16, 0.48) | <0.001 |
| **≥2** **affected vascular systems** | | |
| **LRF** | **Beta (95% CI)** | **p-value** |
| Overweight | 0.64 (0.43, 0.86) | <0.001 |
| Physical activity <1.5h/week | 0.28 (0.09, 0.47) | 0.004 |
| sMDS ≤2 | 0.07 (-0.12, 0.26) | 0.47 |
| Smoking | 0.42 (0.20, 0.65) | <0.001 |

**Table S10:** **Fully adjusted linear regression analysis for the association of each individual LRF with hsCRP according to the extent of ASCVD without multiple imputations**. The regression coefficient (Beta) and the 95% confidence interval (95% CI) are given. HsCRP was log-transformed. Adjustment was made for age, sex, diabetes, arterial hypertension, intake of statins and chronic kidney disease. *ASCVD* atherosclerotic cardiovascular disease; *hsCRP* high-sensitivity C-reactive protein; *LRF* lifestyle-related risk factors; *sMDS* simple mediterranean diet score.
